# PART as a Negative Outcome Modifier of Glioblastoma Treatment, Case Report

**DOI:** 10.3390/neurosci7030053

**Published:** 2026-04-29

**Authors:** Ross Terada, Jennifer Dailey, Sherry Yan, Michael Punsoni, Eric T. Wong

**Affiliations:** 1 Division of Hematology/Oncology, Department of Medicine, Brown University Health, Providence, RI 02903, USA; rterada@brownhealth.org; 2 Division of Neuropathology, Department of Pathology, Brown University Health, Providence, RI 02903, USA; jmingrino@brownhealth.org (J.D.); mpunsoni1@brownhealth.org (M.P.); 3 Department of Radiation Oncology, Boston Medical Center, Boston, MA 02118, USA; sherry.yan2@bmc.org; 4 Department of Neurology, Neurosurgery and Radiation Oncology, Brown University Health, Providence, RI 02903, USA

**Keywords:** glioblastoma, PART, tauopathy, case report

## Abstract

Background: Severe neurocognitive decline is often seen in elderly glioblastoma patients after treatment with radiation and chemotherapy. But the mechanism behind their deterioration is unclear. We describe one such patient with concomitant primary age-related tauopathy (PART) in bilateral hippocampi. Case presentation: An 88-year-old woman experienced unsteadiness, memory loss, and slurred speech that was caused by an epithelioid glioblastoma with wild-type isocitrate dehydrogenase-1 and methylated promoter of *O^6^-methylguanine-DNA methyltransferase*. She was treated with gross total resection, followed by intensity-modulated radiotherapy and daily temozolomide. Shortly after starting treatment, she developed fatigue, anorexia, and neurocognitive impairment, which were refractory to corticosteroids. After two cycles of adjuvant temozolomide, she experienced impulsivity, disorientation, hallucinations, somnolence, and incontinence despite stable neuroimaging findings. Treatment was subsequently discontinued, and she died 20 months from the time of her glioblastoma diagnosis. Autopsy revealed tau-positive neurofibrillary tangles, but rare Aβ plaques, in the trans-entorhinal and entorhinal cortices of both hippocampi. These findings are consistent with a diagnosis of PART. Conclusions: Undiagnosed tauopathy could be a negative modifier of glioblastoma treatment. The identification of PART and other tauopathies as risk factors in the elderly population may be important to guide treatment decision.

## 1. Introduction

Radiation and concomitant radiotherapy are the best available therapeutic modalities to control glioblastoma [1]. Because elderly patients at age 70 or older are particularly susceptible to treatment toxicities, disease management is tailored based on their neurologic performance and *O^6^-methylguanine-DNA methyltransferase* (*MGMT)* promoter methylation status, and consequently they are stratified to receive (i) 3 weeks of concurrent temozolomide and radiation to 4000 cGy in 15 fractions, (ii) radiation alone, or (iii) supportive care [2]. However, this leaves the question of intrinsic vulnerability in this population unanswered.

Primary age-related tauopathy (PART) is a neurodegenerative condition increasingly recognized in cognitively normal elderly individuals when neurofibrillary tangles are found in the medial temporal lobe, basal forebrain, olfactory areas and brainstem [3]. It differs from Alzheimer disease by a lack of concomitant β-amyloid deposition. When PART patients have cognitive decline, their symptoms are milder even when adjusted for age and Braak stage [4]. However, there is no convincing association between tauopathy and glioblastoma, and it is unclear whether tau-related neurodegeneration in the brain is accelerated by treatment with radiation, chemotherapy or both. Here, we describe a patient who experienced subacute neurocognitive decline after treatment for glioblastoma, and PART was diagnosed unexpectedly post mortem.

## 2. Case Description

**Figure 1:**
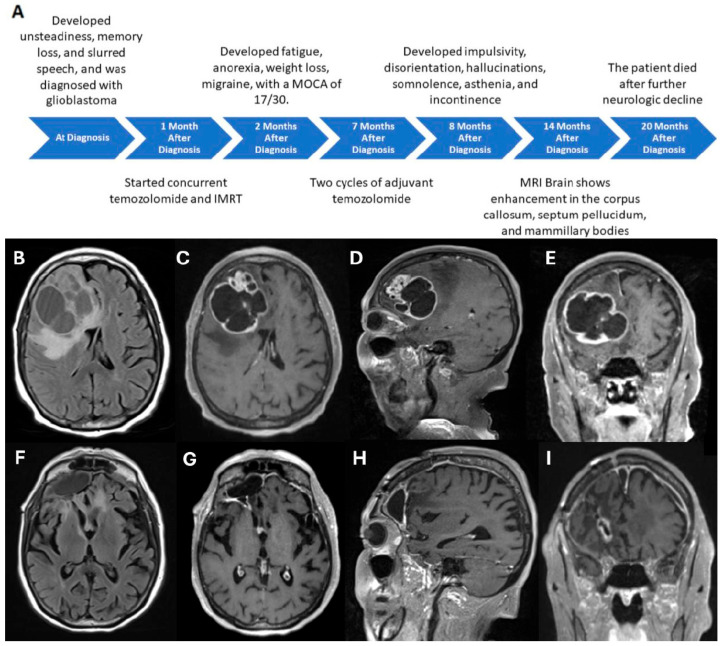
Chronology of clinical progression and MRI findings. (**A**) Timeline depicts the patient’s progressive clinical course (**A**). A multi-cystic enhancing glioblastoma with surrounding vasogenic edema in the right frontal lobe is shown in T2/FLAIR-weighted axial sequence (**B**) as well as post-gadolinium T1-weighted sequences in axial (**C**), sagittal (**D**), and coronal (**E**) views. Stable resection cavity and slight thickening of the septum pellucidum is seen in T2/FLAIR-weighted axial sequence (**F**) as well as post-gadolinium T1-weighted sequences in axial (**G**), sagittal (**H**), and coronal (**I**) views. IMRT: intensity-modulated radiotherapy, MOCA: Montreal Cognitive Assessment.

An 88-year-old woman, with a history of ophthalmic migraine and triple-positive breast cancer, experienced paroxysmal seizures and a cystic heterogeneously enhancing mass was found on MRI in her right frontal lobe. A family history of breast cancer was reported in her paternal grandfather and cousin, together with renal cell carcinoma in her mother. She underwent a gross total resection of the tumor (Figure 1), and pathology revealed a grade 4 epithelioid glioblastoma with wild-type isocitrate dehydrogenase-1 (IDH-1) and methylated promoter of the *MGMT* enzyme, according to the 2021 World Health Organization histological grading criteria. Additional histological features and molecular characteristics from next-generation sequencing of her tumor were previously reported [5]. Briefly, the tumor consisted of two populations of cells, with one having the typical characteristics of glioblastoma accompanied by endothelial proliferation and pseudo-palisading necrosis while the other consisting of larger cells with epithelioid morphology. Immunohistochemistry analysis of both components showed positivity in glial fibrillary acidic protein (GFAP), Olig-2 and Alpha-Thalassemia/Mental Retardation X-linked (ATRX) but negative for IDH-1 and p53, indicating wild-type for both markers. The Ki-67 proliferation rate was approximately 40–50%. Molecular study of the DNA repair enzyme MGMT showed that the promoter region was methylated. Therefore, the histology is consistent with a grade 4 IDH-1 wild-type glioblastoma typically found in the elderly population, but her prognosis may be better than average due to the methylated promoter of *MGMT*.

Whole-exome and whole-transcriptome sequencing were performed. Both populations of the tumor revealed similar genomic signatures and pathogenic variants, harboring identical point mutations in PTEN c.610C>G, RB1 c.55G>T, and TERT promoter c.146C>T, as well as TP53 c.796G>A and c.586C>T. The variant allele frequencies (VAFs) of these common mutations were much higher in the epithelioid component. Two alterations were uniquely identified in the epithelioid component: a nonsense mutation in TAF1 (c.3619C>T, p.R1207*) and a missense mutation in GATA6 (c.874 G>T, p.G292C) genes with a variant allele frequency of 24% and 49%, respectively. Furthermore, electronic karyotype analysis revealed that both populations contained similar copy number alterations involving extra copies of chromosome 7, a deletion of chromosome 16q and a partial deletion of chromosome 10q and 15q, consistent with the molecular data showing copy number gain of EGFR on chromosome 7 (7p11.2) and copy number loss or deletion of PTEN on chromosome 10 (10q23.31). Additional copy number changes were acquired in the epithelioid population, including the gaining of part of chromosome 2q and chromosome 13, the loss of chromosomes 11 and 19q, and additional, more focal alterations in other chromosomes [5].

Human leukocyte antigen (HLA) genotyping and immune cell transcriptome sequencing were performed on both histologic populations of the tumor. No difference was found in major histocompatibility complex (MHC) I and II. However, an analysis of immune cell transcriptome revealed significant differences in their compositions. High levels of myeloid cells, as well as M1 and M2 macrophages, were found in the glioblastoma population, while CD4+ lymphocytes and NK cells were elevated in the epithelioid population. B cells were present at a higher level in the glioblastoma compared to the epithelioid portion of the tumor. Collectively, the pathology data indicate that this tumor has two subpopulations of cells, most likely evolved under different immunologic and selection pressures.

At initial presentation to the Neuro-Oncology Clinic, she was found to have cogwheel rigidity in her wrists, dysdiadochokinesia in her left upper extremity, unsteadiness upon Romberg testing, and poor tandem gait. One of three skin punch biopsies identified α-synuclein aggregates from her upper cervical region, suggesting a possibility of Parkinson’s disease. But a radionuclide neuroimaging study, dopamine active transfer scan (DATscan), showed normal dopamine uptake in the basal ganglia. No family history of neurological, psychiatric or psycho-social disorders was noted.

**Figure 2:**
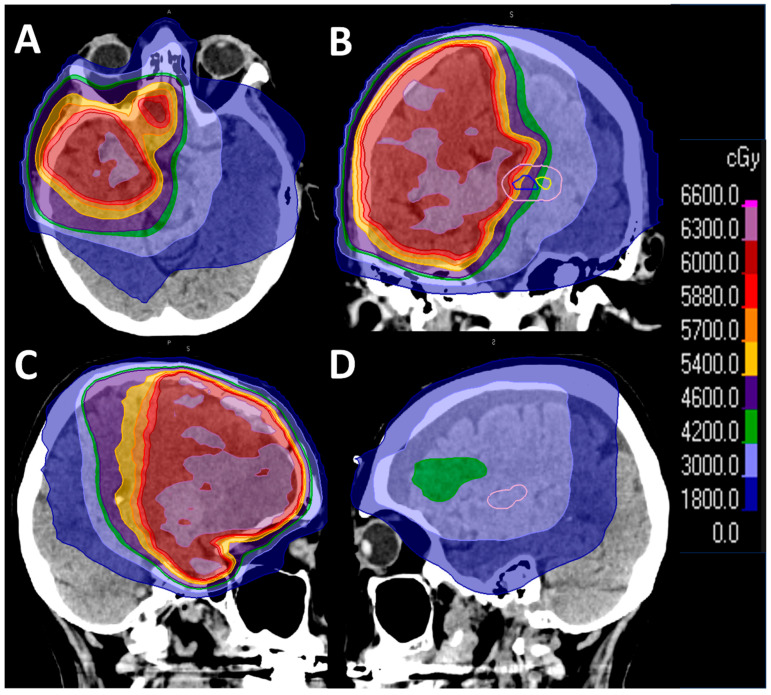
Extensive radiation coverage of both temporal lobes. The left temporal pole and the left hippocampus were exposed to the full dose or 6000 cGy of radiation as shown in axial (**A**), coronal (**B**) and left sagittal (**C**) images. The right temporal pole received between 1800 and 3000 cGy (**D**), while the right hippocampus was exposed to 3000 cGy of radiation (**B**). Small letters within images are A, anterior; P, posterior, and S, superior.

The patient’s glioblastoma was treated with daily temozolomide chemotherapy and intensity-modulated radiation therapy directed at the right frontal brain to a total dose of 6000 cGy in 33 fractions (Figure 2). The 6-week regimen was chosen due to her excellent initial neurologic performance status, and the smaller radiation fraction size was thought to have less neurocognitive sequela compared to the larger fraction size in the 3-week regimen [1,2]. However, the temozolomide had to be discontinued due to thrombocytopenia. She developed extreme fatigue, anorexia, and a 15-pound weight loss. She also experienced her first ophthalmic migraine in ten years. A Montreal Cognitive Assessment (MOCA) [6] performed during this time had a score of 17/30. She was treated with dexamethasone for subacute radiation-induced encephalopathy followed by a 5-month slow taper of the medication. During this period, she developed daily aphasia after physical exertion and had a convulsive seizure once. After completion of radiation and steroid taper, she experienced improvement in her neurological function with independence in activities of daily living and a higher MOCA score of 25/30.

After her recovery, she was treated with two cycles of adjuvant temozolomide at monthly intervals. During her second cycle, she developed increased impulsivity, driving her car to a nearby convenience store against medical advice. She became disoriented to her location and time of day, and experienced visual hallucinations seeing her late husband. Her hallucinations became progressively more persistent, and she developed increased somnolence, generalized weakness, and urinary incontinence. A gadolinium-enhanced head MRI obtained during this period, about 6 months after her second cycle of adjuvant temozolomide, showed hyperintense signals on fluid-attenuated inversion recovery sequence suggesting tumor extension to or the presence of treatment effect at the genu of the corpus callosum, septum pellucidum, and mamillary bodies (Figure 1F). She continued to develop worsening neurologic symptoms and expired four months later, 17 months from the completion of radiation and concomitant temozolomide or 20 months from the initial histologic diagnosis of glioblastoma.

Consent was granted for a full post mortem examination. The brain was fixed in 10% neutral buffered formalin. Macroscopic evaluation of the entire brain and the superior part of the cervical cord revealed a weight of 1071 g (normal: 1250–1450 g). Tissue sections were sampled for microscopic examination of 20 representative sections of the brain and spinal cord including all lobes of the cerebral cortex as well as the basal ganglia, thalamus, amygdala, hippocampus, midbrain, pons, medulla, and cerebellum. Histopathologic examination of hematoxylin and eosin-stained sections was performed on 8-micron-thick sections of formalin-fixed, paraffin-embedded brain tissue. All immunohistochemistry stains were performed on 4-micron-thick sections from the same blocks (see File S1: Supplemental Neuropathology Protocol for additional details on methodology). The neuropathologic diagnosis of PART was determined by an evaluation of neurofibrillary tangles or neurite burden highlighted by tau pathology and by an assessment of plaque morphology (with diffuse plaque morphology in the absence of neuritic plaques) and frequency with β-amyloid [7]. Given the clinical history of glioblastoma, and the morphologic evidence of residual tumor, a GFAP stain was performed to confirm the glial origin of the tumor.

**Figure 3:**
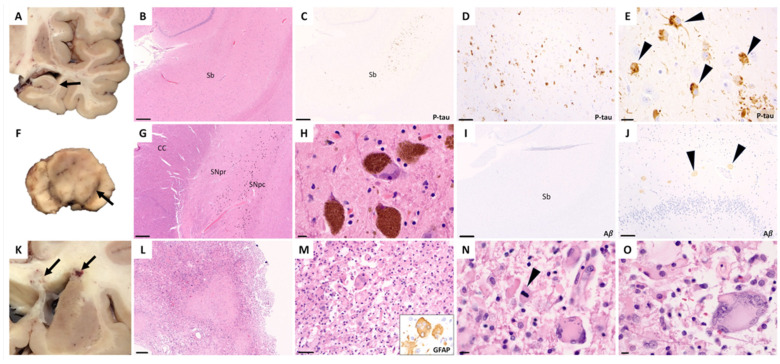
Neuropathology at post mortem. (**A**) Gross image of the right temporal lobe without atrophy (arrow shows the hippocampus). (**B**) Low-power image of the hippocampal formation with intact architecture (scale bar: 500 μm, hematoxylin and eosin). (**C**–**E**) Low-, medium-, and high-power images of phosphorylated tau-stained sections showing p-tau-positive neurofibrillary tangles and neurites (arrowheads) in the subiculum (sb = subiculum; scale bars: 500 μm, 100 μm, 10 μm). (**F**) Gross image of a midbrain cross section shows a well-pigmented substantia nigra (arrow). (**G**,**H**) The substantia nigra pars reticulata (SNpr) and pars compacta (SNpc) demonstrate appropriate neuronal density with neurons containing neuromelanin without evidence of Lewy bodies (CC = crus cerebri; scale bars: 500 μm and 10 μm, hematoxylin and eosin). (**I**,**J**) Staining for β-amyloid (Aβ) in the hippocampal formation shows rare but mild plaques (arrowheads) in CA3 (scale bars: 500 μm and 100 μm). (**K**) Gross image of a coronal section of the brain with tumor (arrows) present in the corpus callosum and right basal ganglia at the level of the dorsal striatum. (**L**,**M**) Residual treated, GFAP+ (glial fibrillary acidic protein, inset) glioblastoma tumor cells with large areas of necrosis (scale bars: 200 μm, 50 μm; hematoxylin and eosin), together with (**N**,**O**) occasional mitoses (arrowhead) and multinucleation (scale bars: 10 μm, 10 μm; hematoxylin and eosin).

Microscopic examination of the brain identified residual glioblastoma tumor cells involving the right frontal lobe, right basal ganglia, corpus callosum, and left basal ganglia, but none in the midbrain, pons, medulla, or spinal cord. The patient was also found within bilateral hippocampi to have tau-positive neurofibrillary tangles involving trans-entorhinal and entorhinal cortices and rare, mild Aβ plaques, consistent with a diagnosis of possible PART, Braak stage III and Thal Aβ phase 2 (Figure 3). The substantia nigra had intact neuronal density and pigmentation, without intracytoplasmic Lewy bodies or α-synuclein positive neurites (Figure 3F–H). Mild to moderate cerebral amyloid angiopathy was seen involving small vessels within the cerebral cortex and leptomeninges.

## 3. Discussion

The finding of neurofibrillary tangles without significant β-amyloid in the entorhinal cortex of our patient is consistent with possible PART, and we postulate that this predisposed her to early subacute neurocognitive decline from radiation and chemotherapy treatments. Whether PART is a stand-alone entity among those minimally affected or part of a spectrum of Alzheimer disease is unclear. Regardless, neurofibrillary tangles are found in both disorders, and pathologic tau is just one of many protein–protein aggregates that are associated with neurodegeneration [3,8]. Lewy bodies, which consist of pathological aggregates of α-synuclein proteins, can also negatively impact treatment outcome in glioblastoma [9]. Therefore, it is prudent for neurologists and oncologists to look for potential signs of neurodegeneration among patients diagnosed with glioblastoma.

Pre-mortem diagnosis of PART, Alzheimer disease or other tauopathies can be specifically established by ^18^F-flortaucipir positron emission tomography (PET) and β-amyloid PET [10,11]. Among patients with tau pathology, it is notable that the presence of β-amyloid predicts a faster longitudinal memory decline compared to those who are β-amyloid negative, consistent with the differences in clinical manifestation between PART and Alzheimer disease [12]. These PET modalities may offer a means to stratify more precisely treatment-related neurocognitive risks among the elderly glioblastoma patients.

Hippocampal neurogenesis is reduced in both aging and after irradiation alone or radiation potentiated by temozolomide. Dysfunctional neurogenesis has been associated with impaired spatial learning and memory in animal models [13,14]. We believe that PART in the entorhinal cortex and hippocampi of our patient may predispose her to radiation toxicity. In humans, whole-brain radiotherapy (WBRT) for brain metastasis results in a higher incidence of mental decline, and the impairment can appear in as early as 3 months after treatment, affecting multiple neurocognitive domains [15]. However, hippocampal avoidance WBRT (HA-WBRT) has been shown to reduce the rate of neurocognitive decline among patients based on their performance on the Hopkins Verbal Learning Test [16]. In glioblastoma, hippocampal avoidance radiotherapy has been proposed but it is limited by inadequate dosing at the margins of the clinical treatment volume where infiltrative tumor cells are located [17]. Although proton radiation is not standard-of-care for invasive glioblastoma, it could spare the hippocampi for the preservation of neurocognitive functions. Furthermore, there is no effective means of clearing tau from the brain and whether an anti-β-amyloid antibody like lecanemab or donanemab can prevent neurocognitive decline from radiation in susceptible individuals is unclear.

The association between tauopathy and glioblastoma is unclear. Lim et al. identified tau hyperphosphorylation and aggregation from glioblastoma-secreted soluble CD44, which is a cell surface glycoprotein involved in cell–cell interaction, adhesion and migration [18]. Because invasion is a major hallmark of glioblastoma [19,20], it is plausible that this tumor may cause tau pathology, at a site far from the epicenter of the tumor like the PART found in our patient. Interestingly, certain microtubule-associated protein tau (*MAPT*) gene isoforms, like *MAPT-201* and *MPAT-205*, are associated with decreased and increased glioblastoma patient survival, respectively [21].

No Lewy bodies or evidence of α-synucleinopathy was found in the substantia nigra or locus coeruleus, and therefore this patient does not have Parkinson’s disease. The presence of α-synuclein aggregates in the subcutaneous nerve endings from the skin punch biopsy, therefore, may be a marker of aging rather than disease [22]. This is because the migration of these pathological aggregates may take years if not decades from the peripheral to the central nervous system.

## 4. Conclusions

In summary, our patient with PART experienced a subacute decline in neurocognitive function after treatment for glioblastoma. The presence of tau and neurofibrillary tangles in the entorhinal cortex could be a negative outcome modifier.

## Data Availability

The original contributions presented in this study are included in the article/Supplementary Materials. Further inquiries can be directed to the corresponding authors.

## Supplementary Materials

The following supporting information can be downloaded at: https://www.mdpi.com/article/10.3390/neurosci7030053/s1. File S1: Supplemental Neuropathology Protocol.

## Abbreviations

The following abbreviations are used in this manuscript:

Aβ	beta amyloid
cGy	centigray
CC	crus cerebri
DaTscan	dopamine active transfer scan
GFAP	glial fibrillary acidic protein
FLAIR	fluid-attenuated inversion recovery
HA-WBRT	hippocampal avoidance WBRT
IDH	isocitrate dehydrogenase
IMRT	intensity-modulated radiotherapy
MGMT	O^6^-methylguanine-DNA methyltransferase
MOCA	Montreal Cognitive Assessment
MRI	magnetic resonance imaging
PART	primary age-related tauopathy
PET	positron emission tomography
SNpc	pars compacta
SNpr	nigra pars reticulata
WBXRT	whole-brain radiotherapy
