# Supplementary material for: PART as a Negative Outcome Modifier of Glioblastoma Treatment, Case Report
Source: NeuroSci. 2026 Apr 29;7(3):53. doi: 10.3390/neurosci7030053 (PMC13214797; doi:10.3390/neurosci7030053)
Supplement: Supplementary file 1 [file neurosci-07-00053-s001.zip › neurosci-4239281-supplementary.pdf]

Supplemental Neuropathology Protocol

Histology

Tissue blocks were processed on a Leica ASP300 using a 20-hour program (fixation with formalin, dehydration with ethanol, clearing with xylene, and infiltration with paraffin wax). This was followed by paraffin-embedding of the tissue to produce a block, which is cut on a Leica RM2255 microtome at 8-microns, mounted on charged Surebond slides (AVANTIK) and stained with hematoxylin-eosin (H&E) on a Tissue Tek Glas (Sakura) Autostainer and coverslipper for standard diagnostic purposes.

Immunohistochemistry

Immunohistochemical studies were conducted on 4-microns-thick sections mounted onto charged slides (AVANTIK) from formalin fixed, paraffin-embedded tissue blocks. The entire immunostaining procedure was performed using a DAKO Autostainer automated staining system. Immunohistochemistry was performed using a mouse monoclonal **β-amyloid** (Aβ) antibody (BioLegend, CA, USA, catalogue number: 800709, clone 4G8) at 1:5000 dilution, a mouse monoclonal anti-glial fibrillary acidic protein (**GFAP**) antibody (BioGenex Laboratories, CA, USA, catalogue number: Mu020-UC, clone GA-5) at 1:2000 dilution, and a rabbit polyclonal anti-tau (**TAU**) antibody (DAKO by Agilent Tech., CA, USA, catalogue number: A0024) at 1:7500 dilution. Detailed antibody procedures are described below.

Procedure for immunostains

- **β-Amyloid** sections were deparaffinized and rehydrated then placed in formic acid for 2 minutes. Sections were stained on the Dako Link 48 Autostainer using the reagents from Dako EnVision FLEX, High pH (link) Kit, catalogue number: K800021-5. Endogenous peroxidase blocking was run for 5 minutes. Beta-Amyloid was incubated for 20 minutes, followed by FLEX HRP incubation for 20 minutes, and DAB is incubation for 10 minutes. The sections were then counterstained with Harris Hematoxylin and cover-slipped off the automatic staining system.
- **GFAP** sections were stained on the Dako Omnis Autostainer using Dako EnVision FLEX, High pH (Dako Omnis) Kit, catalogue number: GV80011-5 with high pH target retrieval and deparaffinization performed on the instrument. Endogenous peroxidase blocking performed for 3 minutes followed by GFAP incubation for 20 minutes. FLEX HRP was incubated for 20 minutes and then DAB was incubated for 5 minutes. Hematoxylin counterstain for 3 minutes.
- **TAU** sections were stained on the Dako Omnis Autostainer using Dako EnVision FLEX, High pH (Dako Omnis) Kit, catalogue number: GV80011-5. Sections were then deparaffinized on the instrument with no Target Retrieval. Endogenous peroxidase blocking was run for 3 minutes. Tau was incubated for 20 minutes, FLEX HRP is incubated for 20 minutes, and then DAB is incubated for 5 minutes. Hematoxylin counterstain for 3 minutes.
